# Dirac operator spectrum in tubes and layers with a zigzag-type boundary

**DOI:** 10.1007/s11005-022-01594-3

**Published:** 2022-10-10

**Authors:** Pavel Exner, Markus Holzmann

**Affiliations:** 1 Doppler Institute for Mathematical Physics and Applied Mathematics, Czech Technical University, Břehová 7, 11519 Prague, Czechia; 2 Department of Theoretical Physics, Nuclear Physics Institute, Czech Academy of Sciences, 25068 Řež near Prague, Czechia; 3 Institut für Angewandte Mathematik, Technische Universität Graz, Steyrergasse 30, 8010 Graz, Austria

**Keywords:** Dirac operator, Zigzag boundary conditions, Spectral analysis, Quantum waveguide, Primary 81Q10, Secondary 35Q40

## Abstract

We derive a number of spectral results for Dirac operators in geometrically nontrivial regions in $\mathbb {R}^2$ and $\mathbb {R}^3$ of tube or layer shapes with a zigzag-type boundary using the corresponding properties of the Dirichlet Laplacian.

## Introduction

In contrast to the Schrödinger case, a physical motivation to study Dirac operators describing particles confined to specific regions was missing for a long time. It appeared for the first time in the 1970s in connection with the attempt to explain the quark confinement by means of the so-called bag models [7, 16]. A different and stronger motivation came three decades later with the discovery of graphene [34]. Although the electrons in a graphene sheet are nonrelativistic, their behaviour can be effectively described by the two-dimensional Dirac equation. Moreover, it appeared that different boundary conditions are of interest in this model depending on the way the graphene specimen is cut from a planar sheet. These applications caused an increasing mathematical interest on these types of Dirac operators, see, e.g. [2, 4, 5, 28, 35] for studies on their self-adjointness and basic spectral properties.

From the mathematical point of view, relations between the shape of the region on which a given operator acts and its spectral properties belong to the most classical questions, and for operators such as the Laplacian there is a huge number of results. The corresponding problem for Dirac operators attracted attention only very recently, cf. [1, 8, 32], and many questions remain open. A particular class of problems concerns a confinement to unbounded regions of a nontrivial geometry. For the Laplacian, or more generally, for Schrödinger operators, it is known that such a confinement can induce a nontrivial discrete spectrum; this problem has been thoroughly analysed. For a broad overview, we refer to the monograph [19] summarizing results of many research papers; particular stronger results will be mentioned in the appropriate places.

It was noted that the spectrum of the two-dimensional Dirac operator with a particular type of boundary conditions, called ‘zigzag’ by the physicists, can be related to the spectrum of the corresponding Dirichlet Laplacian [36]; this fact and its analogue in three dimensions attracted attention recently [14, 24]. The zigzag boundary conditions in a graphene quantum dot emerge from the termination of a lattice, when the direction of the boundary is perpendicular to the bonds. The aim of the present paper is to use these results in combination with the mentioned knowledge about the Laplacian spectra in regions of tube or layer type to derive a number of new spectral results for the corresponding Dirac operators. In the next section, we state in Theorem 2.1 the indicated spectral correspondence and present the needed geometric preliminaries. After that, we will derive our main results for the two- and three-dimensional case in Sects. 3 and 4, respectively. We stress that our aim here is to demonstrate that a number of spectral results for Dirac operators in unbounded regions can be obtained by such a translation, not to strive for stating the conclusions under the weakest possible assumptions. Results on the Laplacian spectrum obtained under weaker hypotheses translate to the Dirac setting analogously.

## Preliminaries

### Definition of the operator and its spectrum

First, let us introduce the Dirac operator with zigzag boundary conditions and let us start with the two-dimensional setting. Denote by $\sigma _1, \sigma _2, \sigma _3 \in \mathbb {C}^{2 \times 2}$ the Pauli spin matrices defined by

2.1 $$\begin{aligned} \sigma _1 = \begin{pmatrix} 0 &{}\quad 1 \\ 1 &{}\quad 0 \end{pmatrix}, \quad \sigma _2 = \begin{pmatrix} 0 &{}\quad -i \\ i &{}\quad 0 \end{pmatrix}, \quad \text {and} \quad \sigma _3 = \begin{pmatrix} 1 &{}\quad 0 \\ 0 &{}\quad -1 \end{pmatrix}. \end{aligned}$$

For $x = (x_1,x_2) \in \mathbb {C}^2$, we will often use the notation $\sigma \cdot x := \sigma _1 x_1 + \sigma _2 x_2$ and, in this vein, $\sigma \cdot \nabla _2 = \sigma _1 \partial _1 + \sigma _2 \partial _2$. Next, let $\Omega \subset \mathbb {R}^2$ be an open set and let $m, c \in \mathbb {R}$ with $m \ge 0$ and $c>0$. Then, the Dirac operator with zigzag boundary conditions is the differential operator $H_{m, \Omega }$ in $L^2(\Omega ; \mathbb {C}^2)$ defined by

2.2 $$\begin{aligned} \begin{aligned} H_{m, \Omega } f&= \big ( -ic \sigma \cdot \nabla _2 + m c^2 \sigma _3 \big ) f = \begin{pmatrix} mc^2 &{}-ic (\partial _1 - i\partial _2) \\ -ic (\partial _1 + i \partial _2)&{} -mc^2 \end{pmatrix} f, \\ \text {dom}\, H_{m,\Omega }&= \left\{ f = (f_1,f_2) \in L^2(\Omega ; \mathbb {C}^2): (\partial _1 + i \partial _2) f_1 \in L^2(\Omega ), f_2 \in H^1_0(\Omega ) \right\} , \end{aligned} \end{aligned}$$

where $H^1_0(\Omega ) = \overline{C^\infty _0(\Omega )}^{\Vert \cdot \Vert _{H^1(\Omega )}}$. We use here the units in which $\hbar =1$, cf. Remark 2.2.

In order to introduce the three-dimensional Dirac operator with zigzag-type boundary conditions, define the Dirac matrices $\alpha _1, \alpha _2, \alpha _3, \beta \in \mathbb {C}^{4 \times 4}$ by

2.3 $$\begin{aligned} \alpha _j = \begin{pmatrix} 0 &{}\quad \sigma _j \\ \sigma _j &{}\quad 0 \end{pmatrix}, \quad j \in \{1,2,3\}, \quad \text {and} \quad \beta = \begin{pmatrix} I_2 &{}\quad 0 \\ 0 &{}\quad -I_2 \end{pmatrix}, \end{aligned}$$

where $\sigma _j$ are the Pauli spin matrices and $I_2$ is the $2 \times 2$-identity matrix. Similarly as above, we use for $x = (x_1, x_2, x_3) \in \mathbb {C}^3$ the notation $\alpha \cdot x = \alpha _1 x_1 + \alpha _2 x_2 + \alpha _3 x_3$ and, in this sense, $\alpha \cdot \nabla _3 = \alpha _1 \partial _1 + \alpha _2 \partial _2 + \alpha _3 \partial _3$ and also sometimes $\sigma \cdot \nabla _3 = \sigma _1 \partial _1 + \sigma _2 \partial _2 + \sigma _3 \partial _3$. Let $\Omega \subset \mathbb {R}^3$ be open. Then, choosing again units such that $\hbar =1$, the three-dimensional Dirac operator with zigzag-type boundary conditions is for $m,c \in \mathbb {R}$ with $m \ge 0$ and $c>0$ the differential operator acting in $L^2(\Omega ; \mathbb {C}^4)$ defined by

2.4 $$\begin{aligned} \begin{aligned} H_{m, \Omega } f&= \big ( -ic \alpha \cdot \nabla _3 + m c^2 \beta \big ) f = \begin{pmatrix} mc^2 I_2 &{}-ic \sigma \cdot \nabla _3 \\ -ic \sigma \cdot \nabla _3 &{} -mc^2 I_2 \end{pmatrix} f, \\ \text {dom}\, H_{m,\Omega }&= \left\{ f = (f_1,f_2) \in L^2(\Omega ; \mathbb {C}^4): (\sigma \cdot \nabla _3) f_1 \in L^2(\Omega ; \mathbb {C}^2),\right. \\&\qquad \left. f_2 \in H^1_0(\Omega ; \mathbb {C}^2) \right\} . \end{aligned} \end{aligned}$$

The basic spectral properties of $H_{m, \Omega }$ in the two- and in the three-dimensional case are summarized in the following theorem. In order to formulate it, we denote by $-\Delta ^\Omega _\mathrm {D}$ the Dirichlet Laplacian in $\Omega \subset \mathbb {R}^d,\, d \in \{2,3\}$.

#### Theorem 2.1

For any $m\ge 0$ and $c>0$, the operator $H_{m,\Omega }$ is self-adjoint. Its spectrum is

$$\begin{aligned} \sigma (H_{m,\Omega }) = \left\{ m c^2 \right\} \cup \left\{ \pm c\sqrt{\lambda + (mc)^2}: \lambda \in \sigma (-\Delta ^\Omega _\mathrm {D}) \right\} \end{aligned}$$

and the following is true:

(i) $$m c^2 \in \sigma _ess (H_{m, \Omega })$$.
(ii) If $m \ne 0$, then $-m c^2 \notin \sigma _p (H_{m, \Omega })$.
(iii) Let $r=1$ for $d=2$ and $r=2$ for $d=3$. For $\lambda > 0$, one has $\pm c \sqrt{\lambda + (mc)^2} \in \sigma _p (H_{m, \Omega })$ with multiplicity *rk* if and only if $\lambda \in \sigma _p (-\Delta _\mathrm {D}^\Omega )$ with multiplicity *k*.

#### Proof

The statements for space dimension $d=3$ follow from [24, Theorem 3.4] noting that the operators $A_m$ in [24] and our $H_{m, \Omega }$ are connected by $H_{m, \Omega } = c A_{mc}$, cf. Remark 2.2.

The statements for $d=2$ follow with the same arguments as in [24, Theorem 3.4]. We also refer to the proof of [14, Theorem 2.4], where the claims are shown in the case of bounded $C^\infty$-domains with compact boundary and $c=1$; for this one has to note that the operator $\mathcal {D}_{0,0, 2}$ in [14] is the orthogonal sum of $\widetilde{H}_{m,\Omega }$ and $H_{m,\mathbb {R}^2 \setminus \overline{\Omega }}$, while $\mathcal {D}_{0,0, -2}$ in [14] is the orthogonal sum of $H_{m,\Omega }$ and $\widetilde{H}_{m,\mathbb {R}^2 \setminus \overline{\Omega }}$, where $\widetilde{H}_{m,\Omega }$ and $\widetilde{H}_{m,\mathbb {R}^2 \setminus \overline{\Omega }}$ are given as in Remark 2.3.

In order to show the claims, introduce in $L^2(\Omega )$ the differential operators $\mathcal {T}_\text {min}$ and $\mathcal {T}_\text {max}$ by

$$\begin{aligned} \mathcal {T}_min f := -i c (\partial _1 - i \partial _2) f, \qquad \text {dom}\, \mathcal {T}_min =H^1_0(\Omega ), \end{aligned}$$

and

$$\begin{aligned} \mathcal {T}_max f := -i c (\partial _1 + i \partial _2) f, \qquad \text {dom}\, \mathcal {T}_max = \left\{ f \in L^2(\Omega ): (\partial _1 + i \partial _2) f \in L^2(\Omega ) \right\} . \end{aligned}$$

Then, as in [36, Proposition 1] one verifies that the operator $\mathcal {T}_\text {min}$ is closed, $\mathcal {T}_\text {max} = \mathcal {T}_\text {min}^*$, and one can write

$$\begin{aligned} H_{m, \Omega } = \begin{pmatrix} mc^2 &{}\quad \mathcal {T}_\text {min} \\ \mathcal {T}_\text {max} &{}\quad -mc^2 \end{pmatrix}. \end{aligned}$$

One finds in the same way as in [24, Proposition 2.2 and Theorem 3.4] that $\mathcal {T}_\text {max} \mathcal {T}_\text {min} = -c^2\Delta ^\Omega _D$ and that $0 \in \sigma _ess (\mathcal {T}_\text {min} \mathcal {T}_\text {max})$; cf. also [36] for similar arguments. Hence, one can apply [24, Proposition A.2] to obtain the claimed results also in the two-dimensional case. $\square$

#### Remark 2.2

The expression of the spectral points in [14, 24] was stated as a mathematical result, with all the physical constants except the mass equal to one. Reintroducing the speed of light, we prefer to use the above indicated form, having in mind that the ‘full’ expression should be

2.5 $$\begin{aligned} \pm \hbar c\sqrt{\left( {\frac{mc}{\hbar }}\right) ^2+\lambda } \end{aligned}$$

which is dimensionally correct; to see that it is sufficient to realize that $\lambda$ has the dimension of inverted squared length, the same as the first term in the square root (recall that $\frac{\hbar }{mc}$ is the Compton wavelength), and the energy of the free nonrelativistic particle confined to the region $\Omega$ with Dirichlet boundary is $\lambda _\mathrm {nr} = \frac{\hbar ^2}{2m}\lambda$.

#### Remark 2.3

In three dimensions, one has $f = (f_1,f_2,f_3,f_4) \in \text {dom}\, H_{m,\Omega } \subset L^2(\Omega ; \mathbb {C}^4)$, formally speaking, if the boundary conditions $f_3|_{\partial \Omega } = f_4|_{\partial \Omega } = 0$ hold, while there are no restrictions to $f_1,f_2$. Let $\widetilde{H}_{m, \Omega }$ be the Dirac operator defined via the formal boundary conditions $f_1|_{\partial \Omega } = f_2|_{\partial \Omega } = 0$, and no restrictions are imposed to $f_3,f_4$. Then, it is not difficult to show that $\widetilde{H}_{m, \Omega }$ is unitarily equivalent to $-H_{m,\Omega }$, so all the results obtained in this paper for $H_{m, \Omega }$ can be translated to corresponding results for $\widetilde{H}_{m, \Omega }$, cf. [24, Lemma 3.2]. We remark that the counterpart of $\widetilde{H}_{m, \Omega }$ in dimension two can be analysed using the same arguments as in the proof of Theorem 2.1 and similar results as in dimension three hold.

### 2D geometry

The object of our interest is spectral properties of $H_{m,\Omega }$ for regions $\Omega$ having the form of tubes and layers. Let us describe their geometry starting from two-dimensional *bent strips* of a fixed width $d=2a$. The key role is played by the strip axis, which is a curve $\Gamma$ of infinite length in $\mathbb {R}^2$ without angles and self-intersections, in other words, graph of a function $\Gamma :\,\mathbb {R}\rightarrow \mathbb {R}^2$ satisfying $|\dot{\Gamma }(s)|=1$ for all $s\in \mathbb {R}$; with an abuse of notation we employ the same symbol for both. We always exclude the trivial case and assume that $\Gamma$ is not a straight line.

At each point of $\Gamma$ we take the segment of the normal of length 2*a* centred at the curve; the strip $\Omega =\Omega _{\Gamma ,a}$ is then the union of these segments. It can be equipped with natural curvilinear coordinates which are the arc length *s* of $\Gamma$ and the normal distance *u* of a strip point from the curve, so that its Cartesian coordinates are

2.6 $$\begin{aligned} x(s,u) = \xi (s)-u\dot{\eta }(s), \quad y(s,u) = \eta (s)+u\dot{\xi }(s), \end{aligned}$$

where dot means derivative with respect to the arc length and the functions $\xi ,\eta$ representing the parametric expression of $\Gamma$ satisfy $\dot{\xi }^2+\dot{\eta }^2=1$. In other words, $\Omega _{\Gamma ,a}$ is the image of a straight strip,

2.7 $$\begin{aligned} \Omega _{\Gamma ,a}:= \big ( x(\Omega _0), y(\Omega _0) \big ), \quad \Omega _0:= \mathbb {R}\times (-a,a). \end{aligned}$$

Using the coordinate functions $\xi ,\eta$ we define the *signed curvature* $\gamma$ of $\Gamma$ by

$$\begin{aligned} \gamma (s) := (\dot{\eta }\ddot{\xi }-\dot{\xi }\ddot{\eta })(s), \end{aligned}$$

which coincides up to the sign with the curvature understood as the inverse radius of the osculation circle to the curve, $|\gamma |=( \ddot{\xi }^2 +\ddot{\eta }^2)^{1/2}$. For bent strips, we assume that

- $\Gamma$ is $C^4$-smooth and the curvature $\gamma$ together with $\dot{\gamma }$ and $\ddot{\gamma }$ tend to zero as $|s|\rightarrow \infty$,
- the map $(x,y):\mathbb {R}\times (-a,a) \rightarrow \Omega _{\Gamma ,a}$ is injective.

Condition (b) means that the strip does not intersect itself; a condition necessary for the injectivity is that $u\gamma (s)>-1$ holds for all $(s,u)\in \mathbb {R}\times (-a,a)$. The curvilinear coordinates *s*, *u* are by construction locally orthogonal: if the above inequality holds for $(s,u)\in \mathbb {R}\times [-a,a]$ and we regard the closure of $\Omega _{\Gamma ,a}$ as a Riemannian manifold with a boundary, its metric tensor is diagonal with the transverse component $g_{uu}=1$ and the longitudinal one $g_{ss}$ equal to $g:=(1+u\gamma )^2$. This also means that the Jacobian of the transformation (2.7) is equal to $\sqrt{g}=1+u\gamma$.

The knowledge of $\gamma$ is crucial because it allows us to reconstruct the curve, uniquely up to Euclidean transformations, and, *mutatis mutandis*, the strip of a fixed halfwidth. Specifically, one uses the quantity $\beta (s_2,s_1):=\,\int _{s_1}^{s_2}\,\gamma (s)\,\mathrm {d}s$ which means the angle between tangent vectors at the respective points of $\Gamma$ to get for a fixed $s_0 \in \mathbb {R}$

2.8 $$\begin{aligned} \xi (s)=\xi (s_0)+ \int _{s_0}^s\cos \beta (s_1,s_0)\,\mathrm {d}s_1, \quad \eta (s)=\eta (s_0)- \int _{s_0}^s\sin \beta (s_1,s_0) \,\mathrm {d}s_1. \end{aligned}$$

Infinite strips of a fixed width are not the only regions in which the relation between the geometry and the spectrum can be studied. A case of interest, for instance, are loop-shaped strips of a fixed width 2*a* built over a Jordan curve of length $L>0$ referring to a map $\Gamma :\,\mathcal {T}\rightarrow \mathbb {R}^2$ where $\mathcal {T}$ is a one-dimensional torus of circumference *L*. Again, we identify $\Gamma$ with an arc length parametrization of the curve. To adapt hypotheses (a) and (b) to this situation, one has to replace $\mathbb {R}$ by the factor space $\mathbb {R}/[0,L)$, or more specifically, to assume that

- for loops: $\Gamma$ is $C^4$-smooth on $\mathcal {T}$ and $\Gamma ^{(k)}(0) = \Gamma ^{(k)}(L)$, $0 \le k \le 4$,
- for loops: the map $(x,y):\mathcal {T}\times (-a,a) \rightarrow \Omega _{\Gamma ,a}$ given by (2.6) is injective.

Returning to infinite strips, we will also consider weak deformations of straight strips of width $d>0$. For simplicity, we study only the case of a one-sided perturbation, i.e. the strip

2.9 $$\begin{aligned} \Omega _{\beta \!f}:= \left\{ \, (x,y)\in \mathbb {R}^2:\, 0<y<d\!+\!\beta f(x)\, \right\} \end{aligned}$$

with the deformation parameter $\beta \ge 0$ and a fixed $f\in C_0^\infty (\mathbb {R})$ with $\text {supp}\, f \subset [-b,b]$.

Finally, we may consider also other types of geometric perturbations, for instance, replacing a smooth bend by a sharply broken or polygonal shape; the simplest example is an L-shaped strip given by

2.10 $$\begin{aligned} \Omega := \left\{ (x,y)\in \mathbb {R}^2: x,y>0,\, \min (x,y)<\pi \right\} . \end{aligned}$$

In such a case the generating curve is polygonal and it makes no sense to ask for smoothness as in (a), while in analogy with (b) we suppose that the strip does not intersect itself. Yet another possibility is to couple different strips together. A notable example is the system of two adjacent strips of widths $d_1,\,d_2$ *coupled laterally* through a ‘window’ of width $\ell =2a$ in the common boundary, i.e. the set

2.11 $$\begin{aligned} \Omega = \big \{ (x,y) \in \mathbb {R}^2: x \in \mathbb {R}, \,y \in (-d_2,d_1) \big \} \setminus \big ( ((-\infty ,-a] \cup [a,\infty )) \times \{ 0 \} \big ). \end{aligned}$$

We denote $d:=\max \{d_1,d_2\}$, $\,D:=d_1+d_2$, and set $\varrho :=d^{-1}\,\min \{d_1,d_2\}$ as the parameter describing the asymmetry of the system.

### 3D geometry

In three dimensions the variety of tubular regions is larger, and in addition, one can investigate spectra of Dirac particles confined to *layers*. As in the previous case, let us start from *bent tubes*. Again, if not mentioned differently we always assume that the generating curve is not a straight line.

Consider an infinite smooth curve $\Gamma$ in $\mathbb {R}^3$ free of self-intersections, in other words, graph of a function $\Gamma :\,\mathbb {R}\rightarrow \mathbb {R}^3$; we again employ the same symbol for both and suppose that $\Gamma$ is parametrized by its arc length, $|\dot{\Gamma }(s)|=1$. An important role in our considerations in the three-dimensional case is played by the Frenet triad frame (*t*, *n*, *b*) which exists whenever $\ddot{\Gamma }(s)\ne 0$; it allows us to introduce curvilinear cylindrical coordinates in the vicinity of $\Gamma$ using the map

2.12 $$\begin{aligned} x(s,r,\theta ):= \Gamma (s) - r\big [ n(s)\cos (\theta -\alpha (s)) + b(s)\sin (\theta -\alpha (s)) \big ], \end{aligned}$$

where $r,\theta$ are the polar coordinates in the plane normal to $\Gamma$ at the point *s* and $\alpha :\mathbb {R}\rightarrow \mathbb {R}$ is a fixed smooth function which describes how the coordinate system (2.12) rotates with respect to the Frenet frame as we move along the curve; as we will see, its choice is important unless the tube considered has a circular cross section. As it is well known, the unit vectors (*t*, *n*, *b*) satisfy, as functions of *s*, the Frenet formula

$$\begin{aligned} \left( \begin{array}{ccc} \dot{t} \\ \dot{n} \\ \dot{b} \end{array}\right) = \begin{pmatrix} 0 &{}\quad \gamma &{}\quad 0 \\ -\gamma &{}\quad 0 &{}\quad \tau \\ 0 &{}\quad -\tau &{}\quad 0 \end{pmatrix} \left( \begin{array}{ccc} t \\ n \\ b \end{array}\right) , \end{aligned}$$

where $\gamma ,\,\tau$ are the curvature and torsion of $\Gamma$, respectively; recall that the knowledge of these functions allows us again to reconstruct the curve uniquely up to Euclidean transformations. We suppose that the curve is sufficiently regular and asymptotically straight,

(c) $\Gamma$ is $C^4$-smooth, $\tau ,\,\dot{\tau },\,\ddot{\tau }$ are bounded, and the curvature $\gamma$ together with its first and second derivatives tend to zero as $|s|\rightarrow \infty$.

The importance of the function $\alpha$ stems from the fact that, in contrast to the two-dimensional situation, the coordinate frame (2.12) need not in general separate the longitudinal and transverse coordinates which is what we need to employ the ‘straightening trick’ similar to that used in the 2D situation. To see the reason, consider first the situation when $\ddot{\Gamma }$ does not vanish anywhere and choose a function $\alpha$ satisfying what the physicists call *Tang condition*,

2.13 $$\begin{aligned} \dot{\alpha }=\tau , \end{aligned}$$

determining the function up to a constant. Under this constraint, the separation occurs. Indeed, regarding the vicinity of $\Gamma$ as a Riemannian manifold, the corresponding metric tensor is

2.14 $$\begin{aligned} (g_{ij})= \left( \begin{array}{ccc} \left( 1+r\gamma \,\cos (\theta \!-\!\alpha )\right) ^2 +r^2(\tau \!-\!\dot{\alpha })^2\; &{}\;0\; &{} \;r^2(\tau \!-\!\dot{\alpha }) \\ 0 &{}\quad 1 &{}\quad 0 \\ r^2(\tau \!-\!\dot{\alpha }) &{}\quad 0 &{}\quad r^2 \end{array} \right) , \end{aligned}$$

cf. [19, Sec. 1.3], becoming diagonal if the condition (2.13) is satisfied. In such a case we have $g:=\det (g_{ij}) = r^2\left( 1+r\gamma \,\cos (\theta \!-\!\alpha )\right) ^2$ and $g^{1/2}$ is the Jacobian of the map (2.12). In general, however, the Frenet triad may not exist globally and/or its local parts may not ‘glue’ together smoothly. This occurs, e.g. when the Frenet triad has one-sided limits at a point $s\in \mathbb {R}$ where (*n*, *b*) are not defined, but those limits do not match. Fortunately, the validity of condition (2.13) is clearly not affected if the function $\alpha$ is shifted by a constant. We thus assume that

(d) the coordinate system (2.12) is *Tang compatible* meaning that (i) the set where $\ddot{\Gamma }(\cdot )$ vanishes consists of isolated zeros accumulating at most at infinity, and (ii) function $\alpha$ is piecewise continuous and such that $\dot{\alpha }(s)=\tau (s)$ holds whenever $\ddot{\Gamma }(s)\ne 0$.

Assumption (d) is convenient to work with, but it should be mentioned that there are interesting examples where it is not satisfied, as the zero set of $\ddot{\Gamma }(\cdot )$ may have a more complicated structure. A particular case of interest is when $\Gamma$ has a straight segment on which its perpendicular parts, (*n*, *b*), are not uniquely defined. The global coordinate system that allows to rephrase the study of $-\Delta _D^\Omega$ as analysis of an elliptic operator on a straight tube does nevertheless exist being obtained by a parallel transport. The existence of such a *relatively parallel adapted frame* for $C^2$-smooth curves was proved in [6] and adapted to the current setting in [27]. In this vein, one can replace assumption (d) by the hypothesis that

(d’) there is a global coordinate system (2.12) in the vicinity of $\Gamma$ separating the longitudinal and transverse coordinates.

For the nontrivial details of assumption (d’), that is only vaguely formulated here, we refer to [27], where it is elaborated for curves $\Gamma$ of an even lower regularity. For us, it is important to remark that the appropriate results in Sect. 4 remain valid when assumption (d) is replaced by assumption (d’).

The importance of these hypotheses follows from the fact that to define a tube built over the curve $\Gamma$ we have to fix its cross section in terms of the transverse part of the coordinates. We suppose that it is an open precompact set $M\subset \mathbb {R}^2$ containing the origin of the coordinates and we set $a:=\sup _{x\in M} |x|$; without loss of generality, we may suppose that *M* is connected. Using the map (2.12), where we identify points in *M* with their polar coordinates, we set

2.15 $$\begin{aligned} \Omega ^\alpha _{\Gamma ,M}:= x(\mathbb {R}\times M), \end{aligned}$$

i.e. we identify the tube with the image of $\Omega _{\Gamma _0,M} := \mathbb {R}\times M$; we will drop the superscript and subscript if they are clear from the context. In order to use map (2.12) to find spectral properties of $H_{m,\Omega }$, we have to assume in addition that

(e) the map *x* in (2.12) is injective,

in other words, that the tube must not intersect itself. It is again not difficult to see that $a\Vert \gamma \Vert _\infty <1$ is sufficient to ensure the injectivity locally, however, its global validity is a stronger requirement. In contrast to the two-dimensional situation, the existence of a locally orthogonal system of coordinates in $\Omega$ requires in view of (2.14) the additional assumption (d) or (d’); assumption (d) can be always satisfied provided the cross section *M* is a disc centred at the origin giving us the freedom to choose the function $\alpha$ with the described license, otherwise it is a restriction to the class of admissible tubes.

The use of assumptions (d) or (d’) does not mean that *noncircular* tubes that do not satisfy it are not of interest; the opposite is true, just they have to be treated by other means. The case of a particular importance concerns *twisted tubes*; for simplicity, we restrict our attention to such tubes built over a straight line. We start again from a straight three-dimensional tube written as a Cartesian product, $\Omega _0= \mathbb {R}\times M$, where the cross section $M\subset \mathbb {R}^2$ has the same properties as before; we exclude the trivial case of a disc. Writing the element of $\mathbb {R}^3$ as a column, $x=(x_1,x_\perp )$, we define the twisted tube as the image of $\Omega _0$ by an appropriate map, namely

2.16 $$\begin{aligned} \Omega _\alpha := \{R_\alpha (x_1)x:\ x \in \mathbb {R}\times M\}, \end{aligned}$$

where $\alpha :\mathbb {R}\rightarrow \mathbb {R}$ is a $C^2$-smooth function with the first and second derivatives bounded on $\mathbb {R}$, and

$$\begin{aligned} R_\alpha (x_1) = \left( \begin{array}{ccc} 1 &{}\quad 0 &{}\quad 0 \\ 0 &{}\quad \cos \alpha (x_1) &{}\quad \sin \alpha (x_1)\\ 0 &{}\quad -\sin \alpha (x_1) &{}\quad \cos \alpha (x_1) \end{array} \right) . \end{aligned}$$

Note that $\Omega _\alpha$ is nothing but the tube discussed above with $\gamma =\tau =0$ and the rotation $\alpha$ with respect to the tube axis not obeying condition (d).

So far we have considered tubes of a fixed cross section. Another interesting situation arises when the latter *varies locally*. Consider a set-valued function $x\mapsto M_x$ which assigns to each $x\in \mathbb {R}$ a precompact, simply connected set $M_x\subset \mathbb {R}^2$ and define

2.17 $$\begin{aligned} \Omega := \bigcup _{x\in \mathbb {R}} \{ x \} \times M_x. \end{aligned}$$

We assume that

(f) $\Omega$ is a local deformation of a straight tube: there is a set $M\subset \mathbb {R}^2$ and $x_0>0$ such that $M_x=M$ if $|x|>x_0$,
(g) the cross section of $\Omega$ varies in a piecewise continuous manner, that is, apart of a discrete set of points, to each $x\in \mathbb {R}$ and $\varepsilon >0$ there is an open set $O\ni x$ such that for any $x'\in O$ the symmetric difference $(M_x\setminus M_{x'})\cup (M_{x'}\setminus M_x)$ is contained in the $\varepsilon$-neighbourhood of the boundary $\partial M_x$. Moreover, the deformation is supposed to obey a global bound: there is a precompact $N\subset \mathbb {R}^2$ such that $M_x\subset N$ holds for all $x\in \mathbb {R}$.

Up to now the generating manifold, the curve over which the tube was built, had codimension two. Let us turn to the situation when the generating manifold is of codimension one, in other words, it is a smooth surface $\Sigma$ in $\mathbb {R}^3$. The task of parametrizing it is now more complicated as there is no natural system of coordinates one could use. In general, one employs an atlas to describe the surface geometry, and even if it consists of a single chart, the existence of a diffeomorphism of $\Sigma$ to the plane expressed in terms of geodesic polar coordinates is not guaranteed [23].

For the presentation simplicity, let us assume for a moment that such coordinates exist, meaning that there is a pole $o\in \Sigma$ such that the exponential mapping $\exp _o: \mathrm {T}_o\Sigma \rightarrow \Sigma$ is a diffeomorphism; if it is not the case, everything can be rewritten in terms of suitable local charts. The ‘radial’ coordinate lines are the geodesics emanating from *o* and the geodesic circles connect points with the same geodesic distance from the pole. The surface $\Sigma$ is expressed by a map $p:\,\Sigma _0\rightarrow \mathbb {R}^3$, where $\Sigma _0:= (0,\infty )\times S^1$ is the plane with polar coordinates, $S^1$ being the unit circle; we write $q=(s,\theta )$. The tangent vectors $p_{,\mu }:=\partial p/\partial q^\mu$ are linearly independent and their cross-product defines a unit normal field *n* on $\Sigma$. This allows us to define locally orthogonal coordinates in the vicinity of $\Sigma$ as the map

2.18 $$\begin{aligned} x(q,u):=p(q)+u n(q), \end{aligned}$$

and the *layer* of width $d=2a>0$ built over the surface $\Sigma$ as the corresponding image of the straight layer $\Omega _0:=\Sigma _0\times (-a,a)$,

2.19 $$\begin{aligned} \Omega := x(\Omega _0). \end{aligned}$$

We assume that the surface is not isomorphic to the *xy* plane and that

(h) the map (2.18) is $C^3$-smooth and injective on $\Omega _0$, i.e. the layer $\Omega$ does not intersect itself. In particular, locally the injectivity requires $\,a<\rho _m:= \left( \max \left\{ \Vert k_1\Vert _\infty , \Vert k_2\Vert _\infty \right\} \right) ^{-1}$, where $k_j$ are the principal curvatures mentioned below, which thus have to be uniformly bounded, $\Vert k_j\Vert _\infty <\infty \,$ for $j=1,2$.

Metric properties of $\Omega$ are derived from those of the generating surface $\Sigma$. Its metric tensor, $g_{\mu \nu }:= p_{,\mu } \cdot p_{,\nu }$, has in the geodesic polar coordinates a diagonal form, $(g_{\mu \nu })=\mathrm {diag}(1,r^2)$, where $r^2\equiv g:=\det (g_{\mu \nu })$ is the square of the Jacobian of the exponential mapping which satisfies the classical Jacobi equation

2.20 $$\begin{aligned} \ddot{r}(s,\theta )+K(s,\theta )\,r(s,\theta )=0 \quad \text {with}\quad r(0,\theta )=1\!-\!\dot{r}(0,\theta )=0, \end{aligned}$$

where $\dot{r}$ denotes the partial derivative of *r* with respect to *s*. The Gauss curvature *K* appearing in (2.20) together with the mean curvature *M* are determined in the usual way: the second fundamental form $h_{\mu \nu }:= -n_{,\mu } \cdot p_{,\nu }$ gives rise to the Weingarten tensor $h_\mu ^{\,\,\nu }:= h_{\mu \rho }g^{\rho \nu }$, where the Einstein summation convention is used at the right-hand side, and it in turn defines the said two curvatures by $K:=\det (h_\mu ^{\,\,\nu })$ and $M:={1\over 2}\mathrm {tr} (h_\mu ^{\,\,\nu })$. What is important for us are the corresponding global quantities obtained by integrating with respect to the invariant surface element, $\mathrm {d}\sigma :=g^{1/2}\mathrm {d}q$, the total Gauss curvature $\mathcal {K}$ and the quantity $\mathcal {M}$, defined, respectively, by

2.21 $$\begin{aligned} \mathcal {K}:=\int _{\Sigma _0} K(q)\, \mathrm {d}\sigma \,, \quad \mathcal {M}:= \left( \int _{\Sigma _0} M(q)^2\, \mathrm {d}\sigma \right) ^{1/2}\,. \end{aligned}$$

The latter always exists, being possibly infinite, while $\mathcal {K}$ requires the integral to make sense which is matter of assumption, cf. (j) below. Recall also that the eigenvalues of the Weingarten map are the principal curvatures $k_1,k_2$ through which the values of the Gauss and mean curvatures are expressed as $K=k_1 k_2$ and $M=\frac{1}{2}(k_1+k_2)$, respectively.

We used the geodetic polar coordinates for illustrative purposes, however, one may consider a much wider class of layers built over a surface $\Sigma$, not necessarily diffeomorphic to a plane, and defined simply as

2.22 $$\begin{aligned} \Omega := \left\{ x\in \mathbb {R}^3:\, \mathrm {dist}(x,\Sigma )<a\right\} \end{aligned}$$

for a given $a>0$. The curvatures mentioned above can be defined globally through local charts of the atlas, which also define a measure on $\Sigma$ that can be used to determine the global quantities of the type (2.21). Assumption (h) is then replaced by the following weaker requirement:

(h’) $\Sigma$ is a $C^2$-smooth, connected and orientable surface embedded in $\mathbb {R}^3$, which is noncompact and complete, i.e. no geodetic on $\Sigma$ is terminated, and the layer built over it does not intersect itself.

In addition to it, we adopt a couple of geometric assumptions:

(i) The layer is asymptotically planar, that is, $\,K(z),\,M(z) \rightarrow 0\,$ for $|z| \rightarrow \infty$ in the sense of the geodetic distance.

If we have the geodesic polar coordinates, this means that $\,K(s,\theta ),\,M(s,\theta ) \rightarrow 0$ holds as $\,s\rightarrow \infty$, however assumption (i) makes sense also if the atlas is more complicated, because the geodetic distance from a fixed point is well defined. Furthermore, we suppose that

(j) the total Gauss curvature exists, $\,K\in L^1(\Sigma _0,\mathrm {d}\sigma )$.

As in the case of tubes, the parametrization using coordinates (2.18) can also be used to describe other curved layers of a fixed width, such as those built over a compact surface without a boundary and periodically curved layers.

In addition to curved fixed-width layers, we can consider flat ones with local deformations such as, for instance,

2.23 $$\begin{aligned} \Omega _f:= \left\{ x=(y,z):\, y\in \mathbb {R}^2,\, 0<z<d+f(y)\right\} , \end{aligned}$$

where $f:\mathbb {R}^2\rightarrow [0,\infty )$ is a bounded function of a compact support, or layers with a two-sided bulge. We have other cases of interest, an important one concerns *laterally coupled layers*. In analogy with the two-dimensional case, by that we mean two adjacent flat layers of the widths $d_1,\,d_2>0$; we suppose that that their common boundary contains a ‘window’ in the form of open set $\mathcal {W}\subset \mathbb {R}^2$, meaning that $\Omega$ has the form

2.24 $$\begin{aligned} \Omega = \big \{ (x,y, z) \in \mathbb {R}^3: x,y \in \mathbb {R},\, z \in (-d_2,d_1) \big \} \setminus \big ( (\mathbb {R}^2 \setminus \mathcal {W}) \times \{ 0 \} \big ). \end{aligned}$$

## The geometrically induced spectrum in two dimensions

After these preliminaries we can describe relations between the spectrum of the operator $H_{m,\Omega }$ and the geometry of the region $\Omega$ that supports it. We consider first the two-dimensional situations and the essential spectrum.

### Theorem 3.1

We have $\sigma _\mathrm {ess}(H_{m,\Omega }) = (-\infty ,-\epsilon _\mathrm {t}] \cup \{mc^2\} \cup [\epsilon _\mathrm {t},\infty )$, where

(i) $\epsilon _\mathrm {t} = c\sqrt{m^2c^2+\big (\frac{\pi }{d}\big )^2}$ if $\Omega$ is a bent strip of width $d=2a$ satisfying assumptions (a) and (b).
(ii) The same is true if $\Omega$ is the weakly deformed strip in (2.9).
(iii) For laterally coupled strips as in (2.11) of the widths $d_1,\,d_2>0$ we have $\epsilon _\mathrm {t} = c\sqrt{m^2c^2+\big (\frac{\pi }{d}\big )^2}$, where $d=\max \{d_1,d_2\}$.

### Proof

To see (i) we note that by [19, Proposition 1.1.1] we have $\sigma _\mathrm {ess}(-\Delta _D^\Omega ) = [\big (\frac{\pi }{d}\big )^2,\infty )$. Hence, Theorem 2.1 implies the claim in (i). Items (ii) and (iii) can be shown in a similar way by using Theorems 1.4 and 1.5 in [19]. $\square$

However, a nontrivial geometry of $\Omega$ can give rise to a nonvoid discrete spectrum of $H_{m,\Omega }$ which is by Theorem 2.1 mirror-symmetric, consisting of eigenvalues $\pm \lambda _j$, supposed to be arranged in the ascending and descending order in the positive and negative part, respectively, multiplicity included.

### Theorem 3.2

Let $\Omega$ be a bent strip of halfwidth *a* satisfying assumptions (a) and (b), then $\sigma _\mathrm {disc}(H_{m,\Omega }) \ne \emptyset$. Furthermore, let $\{\Omega _\beta \}$ be a family of such strips with the curvature equal to $\beta \gamma$ for a fixed function $\gamma$ consistent with (a) and (b) such that $\gamma , \dot{\gamma }, |\ddot{\gamma }|^{1/2} \in L^2(\mathbb {R}, |s| \mathrm {d}s)$; then for all $\beta$ small enough $H_{m,\Omega _\beta }$ has just two simple discrete eigenvalues $\pm \lambda _1(\beta )$ such that

3.1 $$\begin{aligned} \lambda _1(\beta ) = c\sqrt{m^2c^2+\epsilon (\beta )}, \end{aligned}$$

where

3.2 $$\begin{aligned} \sqrt{\big ({\frac{\pi }{2a}}\big )^2\!-\!\epsilon (\beta )}= & {} {\beta ^2\over 8}\left\{ \Vert \gamma \Vert ^2 -\,{1\over 2}\, \sum _{n=2}^{\infty }\, (\chi _n,u\chi _1)^2 \varrho _n \int _{\mathbb {R}^2} \dot{\gamma }(s)\, \mathrm {e}^{-\varrho _n|s-s'|} \dot{\gamma }(s')\, \mathrm {d}s\,\mathrm {d}s' \right\} \nonumber \\&+ \mathcal {O}(\beta ^3), \end{aligned}$$

with $\varrho _n:= \frac{\pi }{2a} \sqrt{n^2\!-\!1}\;$, $\chi _n(u):= \frac{1}{\sqrt{a}}\sin \frac{\pi n}{2a}(u+a)$, and $(\cdot , \cdot )$ being the inner product in $L^2(-a,a)$, the sum runs in fact over even *n* only.

### Proof

In view of Theorem 2.1, one has to establish the existence of a discrete spectrum for the Dirichlet Laplacian on the bent strip which can be done using a variational argument, the idea of which belongs to Goldstone and Jaffe, cf. [22] and [19, Thm. 1.1]. To see the claimed asymptotic expansion, note that $-\Delta _D^\Omega$ has under the stated assumptions by [19, Thm. 6.3] one discrete eigenvalue $\epsilon (\beta )$ which satisfies (3.2). Together with Theorem 2.1 this yields (3.1). $\square$

We mentioned that interesting spectral results can also be obtained for finite strips. A notable example is an isoperimetric-type inequality for loop-shaped strips of halfwidth *a* built over smooth and closed curves without self-intersections of a *fixed length* $L>0$. In that case, the essential spectrum of $H_{m,\Omega }$ consists of a single point, the infinitely degenerate eigenvalue $mc^2$. The rest of the spectrum is purely discrete accumulating only at $\pm \infty$. Asking about optimization of the ‘smallest’ pair of discrete eigenvalues, $\pm \lambda _1$, for all curves satisfying assumptions (a) and (b) for loops, we get the following result:

### Theorem 3.3

In this situation, $\lambda _1$ is uniquely maximized by $\Omega$ in the form of a circular annulus.

### Proof

As the problem reduces by Theorem 2.1 to the maximization of the principal eigenvalue of $-\Delta _\mathrm {D}^\Omega$, the claim follows from Theorem 1, part (a), and the remark afterwards in [18]. $\square$

Theorem 2.1 allows us to get more information about the spectrum of $H_{m,\Omega }$ for infinite curved strips than stated above. For instance, an application of Payne–Pólya–Weinberger inequality proved by Ashbaugh and Benguria [3] yields a lower bound on the distance between $\lambda _1$ and the infinitely degenerate eigenvalue $mc^2$. Obviously, the following bound is the strongest when $H_{m,\Omega }$ has just one pair of discrete eigenvalues.

### Proposition 3.4

Let $\Omega$ be a bent strip of halfwidth *a* satisfying assumptions (a) and (b) and suppose that $\#\sigma _\mathrm {disc}(H_{m,\Omega })=2N$, then we have

3.3 $$\begin{aligned} \lambda _1 \ge c\sqrt{m^2c^2+3^{1-N}b_2\left( {\frac{\pi }{2a}}\right) ^2}, \end{aligned}$$

where $b_2:= \left( \frac{j_{0,1}}{j_{1,1}}\right) ^2 \approx 0.394$ and $j_{r,1}$ is the first zero of the Bessel function $j_{r},\, r=0,1$.

### Proof

By [19, Thm. 3.1] the first eigenvalue of Dirichlet Laplacian in the described situation is bounded from below by $3^{1-N}b_2\big ({\frac{\pi }{2a}}\big )^2$. This and Theorem 2.1 imply the claim. $\square$

In a similar way as above, one can translate to the Dirac operator setting other spectral estimates valid for Dirichlet Laplacians in strips, for instance, the Lieb–Thirring-type inequality for the moments of the sequence $\big \{c\sqrt{\big (\frac{\pi }{2a}\big )^2+(mc)^2}-\epsilon _n\big \}\,$, where $\epsilon _n$ are the nonnegative eigenvalues of $H_{m,\Omega }$, cf. [19, Thm. 3.2].

Note further that the effect of geometrically induced binding we are discussing here is robust; it does not require the strip to have a smooth boundary. As an example, consider an L-shaped strip in (2.10); in the same way as below, one can also translate to the Dirac operator setting the results about spectra of more general polygonal ducts from Section 1.2 of [19].

### Proposition 3.5

Let $\Omega$ be given by (2.10). Then, $\sigma _\mathrm {ess}(H_{m,\Omega }) \!=\! (-\infty ,-c\sqrt{m^2c^2+1}] \cup \{mc^2\} \cup [c\sqrt{m^2c^2+1},\infty )$ and the discrete spectrum consists of a pair of simple eigenvalues, $\pm c\sqrt{m^2c^2+\epsilon _1}$, where $\epsilon _1\approx 0.9291$.

### Proof

According to Theorem 1.2 and Proposition 1.2.3 of [19] applied with $d=\pi$ we have that $\sigma _ess (-\Delta _D^\Omega )=[1,\infty )$ and $-\Delta _D^\Omega$ has one eigenvalue given by $\epsilon _1\approx 0.9291$. Thus, an application of Theorem 2.1 yields the claimed results. $\square$

Another situation where a local modification of a strip geometry may induce the existence of a discrete spectrum arises when the strip is locally modified as in (2.9). A general existence result can be stated in a way independent of the cross section dimension and we state it in the next section, cf. Theorem 4.4. In the two-dimensional situation we are able to demonstrate the following weak deformation behaviour:

### Theorem 3.6

Let $\Omega = \Omega _{\beta f}$ be as in (2.9). For small enough $\beta$, the discrete spectrum of $H_{m,\Omega }$ consists of a pair of simple eigenvalues, $\pm c\sqrt{m^2c^2+\epsilon _1(\beta )}$, provided $\langle f \rangle := \int _\mathbb {R}f(x)\,\mathrm {d}x>0$. They are real-analytic functions at $\beta =0$ satisfying

$$\begin{aligned} \epsilon _1(\beta )= \Big (\frac{\pi }{d}\Big )^2 -\beta ^2 \frac{\pi ^4}{d^2} \langle f \rangle +\mathcal {O}(\beta ^3). \end{aligned}$$

The discrete spectrum is empty if $\langle f \rangle <0$ as well as in the critical case, $\langle f \rangle =0$, if $\beta$ is small and $8b< d\sqrt{3}$. On the other hand, a pair of weak bound states exists for $\langle f \rangle =0$ if

$$\begin{aligned} \frac{\Vert f'\Vert ^2}{\Vert f\Vert ^2}< \frac{24}{9+\sqrt{117\!+\!48\pi ^2}}\, \frac{\pi ^2}{d^2} \end{aligned}$$

and there are positive constants $c_1,\,c_2$ such that

$$\begin{aligned} c_1\beta ^4 \le \Big (\frac{\pi }{d}\Big )^2 - \epsilon _1(\beta ) \le c_2\beta ^4. \end{aligned}$$

### Proof

The results are obtained by Theorem 2.1 and a variational method for analysing the spectrum of the Dirichlet Laplacian in $\Omega$. The first claim follows from Theorem 6.5^1^ of [19] which reproduces the result obtained in [12], the behaviour in the critical case follows from [19, Theorem 6.9]. $\square$

For laterally coupled strips described at the end of Sect. 2.2 we have the following result:

### Theorem 3.7

Let $\Omega$ be given by (2.11). Then, we have $\sigma _\mathrm {disc}(H_{m,\Omega }) \ne \emptyset$ for any $\ell >0$. The eigenvalues $\pm c\sqrt{m^2c^2+\epsilon _j(\ell )}$ are simple with $\epsilon _j(\ell ) \in \big (\big (\frac{\pi }{D}\big )^2, \big (\frac{\pi }{d}\big )^2 \big )$ which are continuously decreasing functions of $\ell$; the number of the discrete eigenvalues of $H_{m, \Omega }$, of both the positive and negative ones, is $2\left( \max \left\{ 1, \left\lfloor {\ell \over d} \sqrt{1\!-\!(1\!+\!\varrho )^{-2}} \right\rfloor \right\} + N\right)$ with $N \in \{0,1\}$. As for the weak-coupling case, there are positive constants $c_1,\,c_2$ such that

$$\begin{aligned} c_1 \ell ^4 \le \left( {\frac{\pi }{d}}\right) ^2- \epsilon _1(\ell ) \le c_2 \ell ^4 \end{aligned}$$

holds for all sufficiently small positive window widths $\ell$.

### Proof

First, it follows from Theorem 1.5 of [19] that $\sigma _disc (-\Delta _D^\Omega )\ne \emptyset$, the same result allows us to estimate positions of the eigenvalues $\epsilon _j(\ell )$ and the critical values of $\ell$ at which eigenvalues emerge from the continuous spectrum. The weak-coupling asymptotics is a consequence of Theorem 6.10 of [19]. The application of Theorem 2.1 yields then the claimed results about the spectrum of $H_{m,\Omega }$. $\square$

Before leaving the two-dimensional case, let us mention one more interesting example. This $\Omega$ consists of two strips, for simplicity both of the width $\pi$, *crossing at the right angle*. In analogy with Proposition 3.5 one can check that $H_{m,\Omega }$ has then a pair of simple eigenvalues $\pm c\sqrt{m^2c^2+\epsilon _1}$, where $\epsilon _1\approx 0.66$. A new feature here is the existence of a pair of eigenvalues $\pm c\sqrt{m^2c^2+\epsilon _2}$ with $\epsilon _2\approx 2.73$ which are embedded in $\sigma _\mathrm {cont}(H_{m,\Omega })$ as one can check using a simple argument combing scaling and symmetry considerations, first proposed in [37].

## The geometrically induced spectrum in three dimensions

Let us pass to the three-dimensional situation, starting again from the essential spectrum of the Dirac particle confined to a tube or layer.

### Theorem 4.1

We have $\sigma _\mathrm {ess}(H_{m,\Omega }) = (-\infty ,-\epsilon _\mathrm {t}] \cup \{mc^2\} \cup [\epsilon _\mathrm {t},\infty )$, where

(i) $\epsilon _\mathrm {t} = c\sqrt{m^2c^2+\mu _1}$, where $\mu _1$ is the principal eigenvalue of the Dirichlet Laplacian $-\Delta _\mathrm {D}^{M}$, if $\Omega$ is a bent tube of cross section *M* satisfying assumptions (c)–(e).
(ii) The same is true if $\Omega$ is a locally deformed tube satisfying assumptions (f) and (g).
(iii) The same is true for the twisted tube (2.16) provided the derivative $\dot{\alpha }$ is compactly supported.
(iv) If, on the other hand, the tube is periodically twisted outside a compact region, $\dot{\alpha }(x_1)=\beta$ for all $|x_1|$ large enough, we have $\epsilon _\mathrm {t} \!=\! c\sqrt{m^2c^2 + \inf \sigma (-\Delta _\mathrm {D}^{M} -\beta ^2\partial ^2_\varphi )}$, where $\varphi$ is the polar angle associated with the transverse variable $x_\perp$.
(v) $\epsilon _\mathrm {t} = c\sqrt{m^2c^2+(\frac{\pi }{d})^2}$ if $\Omega$ is a curved layer of width $d=2a$ satisfying assumptions (h), (i).
(vi) The same is true for the bulged layer (2.23).
(vii) For laterally coupled layers as in (2.24) of the widths $d_1,\,d_2>0$ we have $\epsilon _\mathrm {t} = c\sqrt{m^2c^2+(\frac{\pi }{d})^2}$, where $d=\max \{d_1,d_2\}$.

### Proof

In view of Theorem 2.1, the claims follow from the essential spectrum properties of the corresponding Dirichlet Laplacians. For claim (i), one has by [19, Prop. 1.3.1] that $\sigma _ess (-\Delta _D^\Omega )=[\mu _1, \infty )$, by [19, Thm. 1.4] the same is true under the assumptions in (ii). If the tube is twisted, it is straightforward to check that $-\Delta _\mathrm {D}^\Omega$ is unitarily equivalent to $H_{\dot{\alpha }}:= -\Delta _\mathrm {D}^M - (\dot{\alpha }(x_1)\partial _\varphi +\partial _{x_1})^2$ on $L^2(\Omega _0)$, cf. [19, Sec. 1.7.1]. If $\dot{\alpha }$ is compactly supported, this operator acts as $-\Delta _\mathrm {D}^{\Omega _0}$ outside a compact set and claim (iii) follows. If the tube is periodically twisted, $H_{\dot{\alpha }}$ is unitarily equivalent to the direct integral $\int ^\oplus _\mathbb {R}h(p)\,\mathrm {d}p$, where

4.1 $$\begin{aligned} h(p) = -\Delta _\mathrm {D}^M + (p-i\beta \partial _\varphi )^2 \end{aligned}$$

and $\sigma (H_{\dot{\alpha }}) = \sigma _\mathrm {ess}(H_{\dot{\alpha }}) = [\inf \sigma (h(0)),\infty )\,$ [19, Prop. 1.7.3]. The essential spectrum is preserved under compactly supported perturbations which gives claim (iv).

For curved layers satisfying assumptions (h) and (i) we know from [26] that the essential spectrum of $-\Delta _D^\Omega$ covers the interval $[(\frac{\pi }{2a})^2,\infty )$. For claims (vi) and (vii) we can refer to the proof of Theorem 4.5 in [19] and to [19, Thm. 4.7], respectively. $\square$

What is again more interesting is the discrete spectrum of $H_{m,\Omega }$ induced by the geometry of $\Omega$. If it is nonvoid, by Theorem 2.1 it is mirror-symmetric, $\sigma _\mathrm {disc}(H_{m,\Omega })=\{\pm \lambda _j\}$, where we suppose the positive part to be arranged in the ascending order, multiplicity included.

### Theorem 4.2

Let $\Omega$ be a bent tube of cross section *M* satisfying assumptions (c)–(e), then $\sigma _\mathrm {disc}(H_{m,\Omega }) \ne \emptyset$. Furthermore, let $\{\Omega _\beta \}$ be a family of tubes with the cross section *M* and torsion $\tau$ fixed and the curvature equal to $\beta \gamma$ for a fixed function $\gamma$ consistent with (c) such that $\gamma , \dot{\gamma }, \ddot{\gamma }\in L^1(\mathbb {R}, |s| \mathrm {d}s)$; then for all $\beta$ small enough $H_{m,\Omega _\beta }$ has just two discrete eigenvalues $\pm \lambda _1(\beta )$, each of multiplicity two, such that

4.2 $$\begin{aligned} \lambda _1(\beta ) = c\sqrt{m^2c^2+\epsilon (\beta )}, \end{aligned}$$

where

4.3 $$\begin{aligned} \sqrt{\mu _1-\epsilon (\beta )}\, =\,&\frac{\beta ^2}{8}\Vert \gamma \Vert ^2 - \frac{\beta ^2}{16} \sum _{n=2}^\infty \int _{M\times M} \mathrm {d}y\mathrm {d}y'\, \chi _1(y)\chi _1(y')\chi _n(y)\chi _n(y') \end{aligned}$$

4.4 $$\begin{aligned}&\times \sqrt{\mu _n-\mu _1} \int _{\mathbb {R}^2} h_s(s,y)\,\mathrm {e}^{-\sqrt{\mu _n-\mu _1}|s-s'|}\,h_s(s',y')\, \mathrm {d}s\mathrm {d}s' + \mathcal {O}(\beta ^3). \end{aligned}$$

In this expression $\mu _n$ are the eigenvalues of $-\Delta _\mathrm {D}^{M}$ and $\chi _n$ are the corresponding normalized eigenfunctions; the function $h_s:= r\gamma \tau \sin (\theta -\alpha ) + r\dot{\gamma }\cos (\theta -\alpha )$ is integrated over $\mathrm {d}y= r\mathrm {d}r\mathrm {d}\theta$.

### Proof

By [19, Thm. 1.3], which can be checked by a variational method, one has $\sigma _disc (-\Delta _D^\Omega )\ne \emptyset$. Hence, by Theorem 2.1 also the discrete spectrum of $H_{m, \Omega }$ is nonempty, which shows the first claim. Moreover, for $\Omega = \Omega _\beta$ with sufficiently small $\beta$ the Dirichlet Laplacian $-\Delta _D^{\Omega _\beta }$ has by [19, Thm. 6.3] exactly one simple discrete eigenvalue $\epsilon (\beta )$ that satisfies (4.3). Hence, by Theorem 2.1 (iii) $H_{m, \Omega _\beta }$ has only the pair of discrete eigenvalues, each having multiplicity two, given in (4.2). $\square$

Proposition 3.4 has a three-dimensional analogue:

### Proposition 4.3

Let $\Omega$ be a bent tube of cross section *M* satisfying assumptions (c)–(e) and suppose that $\#\sigma _\mathrm {disc}(H_{m,\Omega })=4N$, then we have

4.5 $$\begin{aligned} \lambda _1 \ge c\sqrt{m^2c^2+3^{1-N}b_3\mu _1}, \end{aligned}$$

where $b_3:= \left( \frac{\pi }{j_{3/2,1}}\right) ^2 \approx 0.489$ with $j_{3/2,1}$ being the first zero of the Bessel function $j_{3/2}$.

The result again comes from Theorem 2.1 and [19, Thm. 3.1]. Other spectral features of Laplacians such as the Lieb–Thirring-type inequality for the moment of the sequence $\{\mu _1-\epsilon _n\}\,$ [19, Thm. 3.2], where $\epsilon _n$ are the nonnegative eigenvalues of $-\Delta _\mathrm {D}^\Omega$, translate to the Dirac operator setting as well.

Passing from curved strips and tubes to straight but locally deformed ones, one can adapt for our purpose Theorem 1.4 of [19] and obtain with Theorem 2.1:

### Theorem 4.4

Let $\Omega$ be the locally deformed tube (2.17) satisfying (f) and (g). The discrete spectrum of $H_{m,\Omega }$ is empty if $M_x\subset M$ for all $x\in \mathbb {R}$. On the other hand, if $M_x\supset M$ for each $x\in \mathbb {R}$ and there is an interval where $M_x\setminus M$ has a nonzero measure, then $\sigma _\mathrm {disc}(H_{m,\Omega }) \ne \emptyset$.

No general existence claim can be made if the deformation of a three-dimensional tube is ‘sign changing’, however, in analogy with the two-dimensional case of Theorem 3.6 one naturally conjectures that for gentle deformations the positivity of the added volume will be decisive for the existence of a mirror pair of bound states of $H_{m,\Omega }$.

Let us turn to twisted tubes. In contrast to bending, twisting gives rise to effective repulsion, and as such stabilizes the spectrum. Theorem 1.8 of [19], based on [17], see also [25], yields in a similar flavour as above the following result which is useful to compare to Theorem 4.2:

### Theorem 4.5

Let $\Omega$ satisfy assumption (c) and (e). Assume further that the cross section *M* has a $C^2$ boundary and is *not* a disc centred at the origin. Moreover, suppose that $\alpha$ in (2.12) is a continuously differentiable function which *violates the condition* $\dot{\alpha }=\tau$ such that $\dot{\alpha }$ is compactly supported, not identically equal to zero and has a bounded derivative. Then, there is an $\varepsilon >0$ such $\Vert \gamma \Vert _\infty + \Vert \dot{\gamma }\Vert _\infty <\varepsilon$ implies $\sigma _\mathrm {disc}(H_{m,\Omega }) =\emptyset$.

By Theorem 4.1(iv) a periodic twist changes the essential spectrum of $H_{m,\Omega }$. Given the fact that the effective repulsion grows stronger with the twist velocity $\beta$, one may expect that a local change of the twist of the correct sign could give rise to the existence of bound states. This is indeed the case. Consider a straight twisted tube (2.16) with the cross section *M* which is not a disc centred at the origin, has a $C^2$-boundary, and moreover,

4.6 $$\begin{aligned} \dot{\alpha }(x_1) = \beta - \delta (x_1), \end{aligned}$$

where $\delta :\mathbb {R}\rightarrow \mathbb {R}$ is a bounded function supported in an interval $[-a,a]$ for some $a>0$.

### Theorem 4.6

In the described situation, $\sigma _\mathrm {disc}(H_{m,\Omega }) \ne \emptyset$ holds provided

4.7 $$\begin{aligned} \int _\mathbb {R}\big (\dot{\alpha }^2(x_1) - \beta ^2)\,\mathrm {d}x_1 < 0. \end{aligned}$$

Moreover, the claim remains valid even when the integral (4.7) vanishes provided that $\dot{\alpha }(x_1) + \beta >0$ holds whenever $|x_1|\le a$ and $\ddot{\alpha }\in L^2(-a,a)$.

### Proof

By Theorems 1.9 and 1.10 in [19], one has under both assumptions in the theorem that $\sigma _disc (-\Delta _D^\Omega )\ne \emptyset$. Hence, by Theorem 2.1 also $\sigma _disc (-H_{m,\Omega })\ne \emptyset$. $\square$

### Remark 4.7

Using the result of [9] one can also prove the absence of the discrete spectrum in the regime opposite to (4.7). A related open question is whether $H_{m,\Omega }$ has resonances corresponding to their Schrödinger counterparts [10, 11].

Let us pass to operators $H_{m,\Omega }$ supported by layers. If $\Omega$ is curved but asymptotically planar, we have the following result:

### Theorem 4.8

Let $\Omega$ be a layer of halfwidth *a* satisfying assumptions (h’)–(j), then $\sigma _\mathrm {disc}(H_{m,\Omega }) \ne \emptyset$ holds if one of the following conditions is satisfied:

(i) The total Gauss curvature satisfies $\mathcal {K}\le 0$. This happens, in particular, if the generating surface $\Sigma$ is not conformally equivalent to the plane so that $\Omega$ is not simply connected.
(ii) The halfwidth *a* is small enough and $\nabla _g M\in L^2_\mathrm {loc}(\Sigma )$, where $\nabla _g$ refers to covariant derivatives on the manifold $(\Sigma ,g)$.
(iii) $$\mathcal {M}=\infty $$ and $$\nabla _g M\in L^2(\Sigma )$$.
(iv) $\Sigma$ has a cylindrical symmetry; if $\mathcal {K}>0$ we have $\#\sigma _\mathrm {disc}(H_{m,\Omega })=\infty$.

### Proof

By Theorem 2.1 the claim is true if we can establish the existence of discrete eigenvalues of $-\Delta _D^\Omega$ under the respective assumptions. In the first three points, this follows from [19, Thm. 4.2] which is based on [13]. In particular, the first one provides a universal existence result for layers which are not simply connected. This follows from the Cohn-Vossen inequality, $\mathcal {K}\le 2\pi (2-2h-e)$, where *e* is the number of ends of $\Sigma$ and *h* is its genus, i.e. the number of ‘handles’; once it is nonzero, we have always $\mathcal {K}<0$, cf. [19, Corollary 4.2.1]. In the last point, the claim is a consequence of Theorems 4.3 and 4.4 in [19] and a corollary of the former. $\square$

### Remark 4.9

(a) There are various other sufficient conditions for the existence of the discrete spectrum of $-\Delta ^\Omega _\mathrm {D}$, see [30] or [33], which ensure in the same way that $\sigma _\mathrm {disc}(H_{m,\Omega })$ is nonvoid. In the same vein, one may ask whether Theorem 2.1 has a higher-dimensional analogue which could be used in combination with the results of [29, 31].

(b) The trial functions used to establish claim (iv) of the last theorem have compact supports which can be chosen arbitrarily far from the symmetry axis. This shows that the result remains valid if such a cylindrical layer is locally deformed. An example is a *conical layer*, cf. [19, Example 4.2.3] and [20].

The claim (iv) of Theorem 4.8 shows an important difference between curvature-induced bound states in tubes and layers. The former case is of a local character, while for layers the global geometry plays a role. This is connected with the fact that a tube can fully be ‘straightened’ using curvilinear coordinates (2.12), while in layers the metric tensor of $\Sigma$ remains always present. As a consequence, for instance, it is not guaranteed that a ‘gentle’ perturbation will give rise to a single bound state of $-\Delta ^\Omega _\mathrm {D}$ as we recalled in the previous remark. From this reason, we restrict our attention to locally curved layers for which $\mathcal {K}=0$ and consider, e.g. the family of surfaces

4.8 $$\begin{aligned} \Sigma _\beta := p(\mathbb {R}^2),\qquad p(x;\beta ) = \big (x,\beta f(x)\big ), \end{aligned}$$

where $f:\mathbb {R}^2\rightarrow \mathbb {R}$ is a given $C^4$-smooth function. For the sake of simplicity, we suppose that *f* is zero outside of a compact set; the conclusions extend to situations where *f* together with its derivatives up to the fourth order has suitable decay properties at infinity [19, Thm. 6.4]. We have the following claim:

### Theorem 4.10

Let $\{\Omega _\beta \}$ be a family of layers of halfwidth *a* built over the surfaces (4.8). Then, $H_{m,\Omega _\beta }$ has for all $\beta$ small enough exactly one pair of discrete eigenvalues $\pm \lambda _1(\beta )$, each having multiplicity two, behaving as

4.9 $$\begin{aligned} \lambda _1(\beta ) = c\sqrt{m^2c^2+\left( {\frac{\pi }{2a}}\right) ^2 -\mathrm {e}^{2w(\beta )^{-1}}}, \end{aligned}$$

where

4.10 $$\begin{aligned} w(\beta ) = -\beta ^2 \sum _{n=2}^\infty (\chi _1, u\chi _n)^2 \Big (\frac{\pi }{2a}\Big )^4 (n^2-1)^2 \int _{\mathbb {R}^2} \frac{|\hat{m}_0(\omega )|^2}{|\omega |^2 + \big (\frac{\pi }{2a}\big )^2 (n^2-1)}\, \mathrm {d}\omega + \mathcal {O}(\beta ^3)\,; \end{aligned}$$

here $\{\chi _n\}$ are the normalized eigenfunctions of the Dirichlet Laplacian on $(-a,a)$, moreover, $(\cdot , \cdot )$ is the inner product in $L^2(-a,a)$ and $\hat{m}_0$ is the Fourier image of $m_0:=\frac{1}{2}\Delta f$.

### Remark 4.11

Returning to the dimensional consideration of Remark 2.2 we note that $w(\beta )$ is dimensionless as it should be. On the other hand, Theorem 6.4 of [19] on which we rely is again stated as a mathematical result; properly speaking the last term in the square root of (4.9) should read $-\frac{1}{L^2}\,\mathrm {e}^{2w(\beta )^{-1}}$, where *L* is the quantity fixing the length scale. This does not change the principal conclusion, namely that the weak-coupling behaviour of these bound states is the same as for two-dimensional Schrödinger operators, with an exponentially small gap. The same conclusions can be made about the weak-coupling gap in Theorems 4.14 and 4.15.

### Proof of Theorem 4.10

According to [19, Thm. 6.4] the discrete spectrum of $-\Delta _{\Omega _\beta }$ consists under the given assumptions of one simple eigenvalue $\big ({\frac{\pi }{2a}}\big )^2 -\mathrm {e}^{2w(\beta )^{-1}}$ with $w(\beta )$ satisfying (4.10). This and Theorem 2.1 (iii) imply the claim. $\square$

In contrast to the two-dimensional situation little is known about spectral properties of sharply broken layers. Using the result of [15] we get in a similar way as above a result about the ‘octant’, or ‘Fichera’ layer, which can be regarded as a counterpart to the L-shaped strip, namely the region

$$\begin{aligned} \Omega := \{(x,y,z)\in \mathbb {R}^2: x,y,z>0,\, \min (x,y,z)<\pi \}. \end{aligned}$$

### Proposition 4.12

One has $\sigma _\mathrm {ess}(H_{m,\Omega }) = (-\infty ,-c\sqrt{m^2c^2+\epsilon _\infty }] \cup \{mc^2\} \cup [c\sqrt{m^2c^2+\epsilon _\infty },\infty )$, where $\epsilon _\infty \approx 0.93$ refers to the L-shaped planar strip of the width $\pi$ in Proposition 3.5. The discrete spectrum of $H_{m,\Omega }$ consists at most of a finite number^2^ of eigenvalues of the form $\pm c\sqrt{m^2c^2+\epsilon _j}$ with $\epsilon _j\in (0,\epsilon _\infty )$.

The isoperimetric-type inequality of Theorem 3.3 has a three-dimensional analogue. Consider a layer of a fixed halfwidth *a* built over a compact surface $\Sigma$ without a boundary. The essential spectrum of $H_{m,\Omega }$ consists again of a single point, the infinitely degenerate eigenvalue $mc^2$, and the rest of the spectrum is purely discrete accumulating only at $\pm \infty$. We take the family of all such layers of halfwidth *a* satisfying assumption (h) and such that the area of the surface $\Sigma$ is fixed, and ask about optimization of the ‘smallest’ pair of eigenvalues, $\pm \lambda _1$.

### Theorem 4.13

In this situation, $\lambda _1$ is uniquely maximized by layers built over a spherical $\Sigma$.

### Proof

What matters is again by Theorem 2.1 the principal eigenvalue of $-\Delta _\mathrm {D}^\Omega$, and therefore, the claim follows from Theorem 1, part (b), in [18]. $\square$

Returning to infinite layers, consider now locally deformed ones of the type (2.23) with a compactly supported function $f \in C_0^\infty (\mathbb {R}^2)$. As before a local protrusion creates bound states:

### Theorem 4.14

Let $f\ge 0$. If there is an $\eta >0$ and an open $\mathcal {W}\subset \mathbb {R}^2$ such that $f(x)>\eta$ for $x\in \mathcal {W}$, $\,\sigma _\mathrm {disc}(H_{m,\Omega }) \ne \emptyset$. Moreover, if *f* is replaced by $\beta f$ with *f* not necessarily positive, but such that $\langle f\rangle := \int _{\mathbb {R}^2} f(x)\,\mathrm {d}x>0$, the operator $H_{m,\Omega }$ has for all sufficiently small $\beta >0$ just one pair of discrete eigenvalues, $\pm \lambda _1(\beta )$, each having multiplicity two, and

4.11 $$\begin{aligned} \lambda _1(\beta ) = c\sqrt{m^2c^2+\left( {\frac{\pi }{d}}\right) ^2 -\mathrm {e}^{2w(\beta )^{-1}}},\qquad w(\beta ) = -\beta \, \frac{\pi }{d^2}\,\langle f\rangle + \mathcal {O}(\beta ^2). \end{aligned}$$

### Proof

First, by [19, Theorems 4.5] one has $\sigma _disc (-\Delta _D^\Omega ) \ne \emptyset$, which implies with Theorem 2.1 (iii) that $\,\sigma _\mathrm {disc}(H_{m,\Omega }) \ne \emptyset$. To see the second claim, we note that by Theorem 6.6^3^ in [19] the operator $-\Delta _D^\Omega$ has, for sufficiently small $\beta$, exactly one discrete eigenvalue

$$\begin{aligned} \epsilon (\beta ) = \left( {\frac{\pi }{d}}\right) ^2 -\mathrm {e}^{2w(\beta )^{-1}} \end{aligned}$$

with $w(\beta )$ given as in (4.11). Hence, Theorem 2.1 implies also the second claim. $\square$

Let us finally consider the laterally coupled layers. The essential spectrum of them is given by Theorem 4.1(vii). In this case, the existence of a discrete spectrum is easy to establish but the weak-coupling result is less precise than in the previous cases:

### Theorem 4.15

Let $\mathcal {W}$ be an open bounded set and let $\Omega$ be defined by (2.24) with $d=\max \{d_1,d_2\}$; whenever $\mathcal {W}$ is nonempty, $\,\sigma _\mathrm {disc}(H_{m,\Omega }) \ne \emptyset$. Moreover, if $\mathcal {W}=\beta M$ with *M* open and nonempty, the operator $H_{m,\Omega }$ has for all sufficiently small $\beta >0$ just one pair of discrete eigenvalues, $\pm \lambda _1(\beta )$, each having multiplicity two, and there are positive $c_1,\,c_2$ such

4.12 $$\begin{aligned} \mathrm {e}^{-c_2\beta ^{-3}} \le c\sqrt{m^2c^2 + \left( \frac{\pi }{d}\right) ^2} -\lambda _1(\beta ) \le \mathrm {e}^{-c_1\beta ^{-3}}. \end{aligned}$$

### Proof

First, by [19, Theorem 4.7] one has $\sigma _disc (-\Delta _D^\Omega ) \ne \emptyset$. Moreover, if $\mathcal {W}=\beta M$ with *M* open and nonempty, then [21, Theorem 3.1] implies that for sufficiently small $\beta >0$ there is only one discrete eigenvalue $\epsilon (\beta )$ of $-\Delta _D^\Omega$ that satisfies

$$\begin{aligned} \mathrm {e}^{-c_2\beta ^{-3}} \le \left( \frac{\pi }{d}\right) ^2 -\epsilon (\beta ) \le \mathrm {e}^{-c_1\beta ^{-3}}. \end{aligned}$$

Hence, all claims in the theorem follow from Theorem 2.1 (iii). $\square$

## Data Availability

Data sharing is not applicable to this article as no datasets were generated or analysed during the current study.

## Footnotes

1 This result in [19] contains a misprint, $\langle f\rangle$ has there an extra square.

2 In [15] a numerical argument is used to show that the discrete spectrum is nonempty.

3 This result in [19] contains the same misprint as [19, Theorem 6.5], an extra square of $\langle f\rangle$.
